# Niemann-Pick Disease Type C (NPDC) by Mutation of *NPC1* and *NPC2*: Aberrant Lysosomal Cholesterol Trafficking and Oxidative Stress

**DOI:** 10.3390/antiox12122021

**Published:** 2023-11-21

**Authors:** Dongun Lee, Jeong Hee Hong

**Affiliations:** Department of Health Sciences & Technology, GAIHST, Gachon University, 155 Getbeolro, Yeonsu-gu, Incheon 21999, Republic of Korea; sppotato1@gmail.com

**Keywords:** cholesterol trafficking, Niemann-Pick disease type C, lysosomal storage disorders, lysosomal proteins, oxidative stress

## Abstract

Cholesterol trafficking is initiated by the endocytic pathway and transported from endo/lysosomes to other intracellular organelles. Deficiencies in cholesterol-sensing and binding proteins NPC1 and NPC2 induce accumulation in lysosomes and the malfunction of trafficking to other organelles. Each organelle possesses regulatory factors to induce cholesterol trafficking. The mutation of *NPC1 and NPC2* genes induces Niemann-Pick disease type C (NPDC), which is a hereditary disease and causes progressive neurodegeneration, developmental disability, hypotonia, and ataxia. Oxidative stress induces damage in NPDC-related intracellular organelles. Although studies on the relationship between NPDC and oxidation are relatively rare, several studies have reported the therapeutic potential of antioxidants in treating NPDC. Investigating antioxidant drugs to relieve oxidative stress and cholesterol accumulation is suggested to be a powerful tool for developing treatments for NPDC. Understanding NPDC provides challenging issues in understanding the oxidative stress–lysosome metabolism of the lipid axis. Thus, we elucidated the relationship between complexes of intracellular organelles and NPDC to develop our knowledge and suggested potential antioxidant reagents for NPDC therapy.

## 1. Introduction

Niemann-Pick type C (NPC) is a lysosomal membrane-bound protein that maintains lipid homeostasis by mediating cholesterol trafficking from lysosomes [1]. The critical component of cellular membranes, cholesterol, has to be transported by binding proteins because cholesterol has hydrophobicity, which disturbs transportation through water [2].

**Figure 1:**
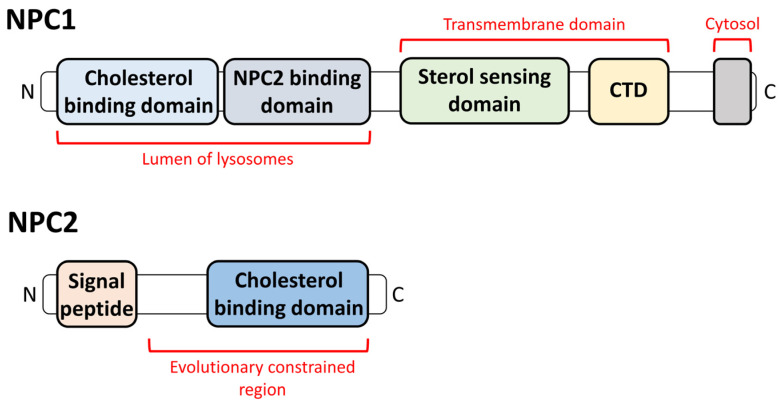
The protein structures of NPC1 and NPC2. The NPC1 has cholesterol/NPC2 binding sites in lysosomal lumen and sterol sensing domain/C terminal domain (CTD) in NPC1 transmembrane site. The NPC2 has signal peptides and cholesterol-binding domain.

Both NPC1 and NPC2 interact with cholesterol for transportation [3]. NPC1 is ubiquitously expressed, and cholesterol is transferred from NPC2 to NPC1 [4]. Structurally, NPC1 is a large glycoprotein containing a transmembrane domain and a sterol-sensing domain [5,6]. NPC1 is located on the transmembrane of lysosomes, while NPC2 is located in the lysosomal lumen to bind cholesterol [7], which is generated by the cholesteryl ester of low-density lipoprotein (LDL) hydrolysis in late endosomes and lysosomes [8,9]. NPC2-bound free cholesterol is transferred to the *N*-terminal domain of NPC1 for cholesterol transportation [10,11,12] (Figure 1).

Cholesterol is a main component of the cellular membrane and is acquired for the formation of LDL to bind with the LDL receptor. Specifically, for circulation in blood vessels, cholesterol is esterified to form lipoproteins, including LDL [13]. The circulating LDL binds with the LDL receptor on the cell surface to internalize into the cells [14]. The LDL passes through early endosomes, late endosomes, and lysosomes sequentially [14]. In lysosomes, the LDL is hydrolyzed to be converted into an unesterified form, which is also known as free cholesterol [14]. Then, NPC2 binds free cholesterol and transfers free cholesterol to NPC1 for transportation toward other intracellular organelles, including Golgi, ER, mitochondria, and the extracellular matrix (through exocytosis) [14,15].

NPC proteins regulate cholesterol trafficking from lysosomes to other cellular organelles and the extracellular matrix. Thus, the malfunction of NPC proteins disturbs cholesterol transportation. Pathologically, mutations of the *NPC1* and *NPC2* genes induce the fetal inherited disorder Niemann-Pick disease type C (NPDC), wherein cholesterol aberrantly accumulates in lysosomes [16]. The accumulation of cholesterol in NPDC triggers lysosomal storage disorders (LSDs) with the accumulation of sphingomyelin in various organs, including the liver, spleen, and brain [1]. LSDs include 70 different heritable diseases, including NPDC [17], and LSDs accompany progressive neurodegeneration, which is suggested to be a hallmark of LSDs [18]. Various clinical symptoms, such as hydrops fetalis [19], dysmorphism [20], organomegaly [21], hypertonia [22], irritability [22], twitching [22], joint swelling [23], cardiomyopathy [24], neonatal liver disease [25], skeletal dysplasia [26], and heart disease, accompany LSDs [27]. In addition, the NPDC has various manifestations, including hepatosplenomegaly, jaundice, and pulmonary infiltration [28]. Cholesterol-lowering treatment and recovery of the *NPC* mutation through transgene therapy are suggested as treatments for NPDC [29]. For the diagnosis of NPDC, patients require the detection of various biomarkers, including oxysterols, lysosphingolipids, and 3β,5α,6β-trihydroxy-cholanoyl-glycine in bile acids [30]. In addition, the specimen from NPDC patients is stained with filipin to evaluate the cholesterol deposits [30]. Recently, molecular genetic methods, including traditional gene sequencing and next-generation sequencing, have been used for NPDC diagnosis [30]. 

Along with pathological lesions, NPDC leads to oxidative stress-mediated cell death [31,32]. The treatment of an in vitro NPDC model with the cholesterol transport inhibitor U18666A increased c-Abl/p73-induced pro-apoptotic signaling, and the reactive oxygen species (ROS) scavenger *N*-acetylcysteine attenuated the expression of c-Abl and p73 [33]. Additionally, intracellular cholesterol trafficking-related organelles, including lysosomes, the ER, Golgi, and mitochondria, are involved in oxidative stress-mediated responses and NPDC [31,34,35,36,37,38,39,40,41]. For example, vitamin E, an antioxidant molecule [42,43], accumulated in the lysosomes of NPDC mice [44]. The accumulation of vitamin E induced an imbalance in ROS, which has the potential to produce oxidative stress [44]. In addition, the mitochondrial activity is related to oxidative stress. The levels of adenosine triphosphate (ATP), which is the product of mitochondrial oxidative phosphorylation, were decreased in the brain, muscle, and liver of NPDC mice [45]. NPDC induces malfunctions in mitochondria, with the potential to cause imbalances in ROS levels [45]. Accordingly, the investigation of physiological roles between NPDC and intracellular organelles could help understand oxidative mechanism-associated NPDC. This review focused on the basic mechanisms of each intracellular organelle, the details of mechanisms related to oxidative stress in NPDC, and suggested antioxidants as NPDC drugs.

## 2. The Relationship between NPDC and Lysosomes

Cholesterol balance is modulated in the lysosomal fraction. Lysosomes have numerous proteins to control lysosomal activity, including the activation of hydrolase, the stimulation of autophagy, and overloading cargo [46,47]. The dominant function of lysosomes is the regulation of protein and lipid contents through the degradation process [48]. The acidic lysosomal lumen is required for lysosomal activity, also called lysosomal clearance, to degrade the proteins. In addition to protein clearance, the cholesterol content is directly modulated by lysosomal membrane proteins. Understanding lysosomal activation and cholesterol trafficking is required to develop NPDC treatment. Thus, we highlighted the relationship between NPDC and the lysosomal proteins and modifications, including NPC proteins, and summarized the current therapies against NPDC.

### 2.1. Lysosomal Appearance and the Changes in Lysosomes in NPDC

**Figure 2:**
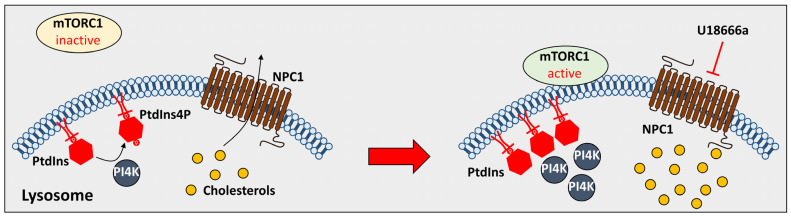
The effect of U18666a on PtdIns4P. The treatment of U18666a inhibits phosphorylation of PtdIns and induces activation of mTORC1 to inhibit cholesterol transport. NPC1: Niemann-Pick type C 1; PtdIns4P: phosphatidylinositol 4-phosphate; and PI4K: phosphatidylinositol 4 kinases.

Lysosomes are complicated intracellular organelles regulated by luminal pH, cellular nutrients, protein homeostasis, the transportation of ions, and pathogenic infections [47,49,50,51]. NPDC and cholesterol accumulation induce imbalances in cellular functions. To maintain the function of lysosomal membrane proteins, lysosomes must exist in intact forms to not cause membrane destabilization. *NPC1* knock-out disrupted lysosomal morphology in a mice model, and increased lysosomal-associated membrane protein (LAMP)-1 was observed in NPDC mice and NPDC patients [52,53,54]. LAMP-1 is expressed in the lysosomal membrane to modulate lysosomal activity. Deficiencies in *NPC1* not only increased LAMP-1 expression but also LAMP-1 glycosylation [55]. Glycosylation is an essential process for lysosomal membrane proteins for vesicle trafficking, and the abnormal glycosylation of LAMP-1 possesses the potential to induce NPDC [56]. LAMP-1 hyper-glycosylation was observed in the cerebellum of *NPC1* knock-out mice [55]. In addition, lysosomal membrane stabilization is associated with ion transportation through the lysosomal membranes. The transportation of Ca^2+^ has been fully studied for lysosomal activation [57]. Glycyl-L-phenylalanine 2-naphthylamide (GPN, lysosomotropic agent, a drug that penetrates lysosomes)-induced lysosomal Ca^2+^ release was attenuated in an NPDC-mimicking cellular model using U18666a with a megakaryocyte cell line (MEG-01), as well as in bacterial-infected RAW 264.7 cells, which inhibited the expression of NPC1 [52,58]. Regardless of the lysosomal components, the activation of lysosomes is regulated by lysosomal positioning [59]. Lysosomal movement plays a role in degrading substrates by fusing with autophagosomes and delivering cargos such as cholesterol. However, cholesterol accumulation in NPDC blocks lysosomal positioning [60]. In lysosomal transportation of cholesterol to the ER, the phosphorylation of phosphatidylinositol (PI) through phosphatidylinositol 4 kinases (PI4Ks) is induced to synthesize phosphatidylinositol 4-phosphate (PtdIns4P), which is located on the lysosomal membrane [61]. The inhibition of NPC1 through U18666a recruited PtdIns4P and PI4K and accumulated PtdIns4P and PI4K in the lysosomal membrane to inhibit autophagy by activating rapamycin complex 1 (mTORC1), as described in Figure 2 [61]. Consequently, deficiency in NPC proteins modulates lysosomal proteins and positioning and subsequently blocks lysosomal cholesterol trafficking. We have summarized the details of the NPDC effect on lysosomes (Table 1).

### 2.2. The Effect of the Lysosomal Proteins on NPDC

Lysosomal membrane proteins have connections with ER membrane proteins to transfer cholesterol from lysosomes to the ER. The ER is associated with lysosomal membrane proteins, such as star-related lipid transfer domain-containing 3 (StARD3), ras-related protein rab-7, and oxysterol-binding protein homologue express [81]. In *NPC1* mutant Chinese hamster ovary (CHO) cells, endolysosomal protein annexin A6 blocks ER-endolysosome connection by inhibiting StARD3 and rab-7 [82]. The knock-down of *annexin A6* in *NPC1* mutant CHO cells recovers cholesterol trafficking endolysosomes to ER [82]. In addition, the motor protein of lysosomes affects cholesterol transportation. For example, the biogenesis of lysosome-related organelle (BOLC) one-related complex (BORC) recruits ARL8 to bind the kinesin light chain/kinesin family member 5 complex, which binds microtubules to move lysosomes [83]. The knock-out of *BORC* and *ARL8* caused cholesterol accumulation, which is indicated by an increase in the filipin intensity in HeLa cell lysosomes [74]. Disruption of the BORC/ARL8 complex induced the secretion of NPC2 from lysosomes [74]. Lysosomes have components that are physically involved in the transportation of cholesterol. The lysosomal proteins not only directly contact proteins but are involved in cholesterol transportation and NPDC-related reactions. Nutrient starvation is the classical stimulation of macroautophagy, which induces lysosomal activation by decreasing amino acid levels in lysosomes [84]. This starvation inhibits mammalian targets of mTORC1, which inhibits lysosomal binding to lysosomes [85]. mTORC1 inhibition induced nuclear translocation of the transcription factor EB (TFEB) to express lysosomal genes to activate lysosomes [85]. The knock-out of *NPC1* induced mitochondrial damage and lysosomal membrane damage with the hyper-activation of mTORC1 in mouse embryonic fibroblasts [75]. The deletion of *NPC1* disrupted mitochondria morphology and increased LAMP2 accumulation [75]. The inhibition of mTORC1 through the Torin1 mTORC1 inhibitor recovered mitochondrial function and lysosomal membranes [75]. The TFEB activator genistein stimulated nuclear translocation and autophagic flux to increase LC3 II expression in NPDC patient fibroblasts [76]. Treatment with genistein induced lysosomal exocytosis and decreased cytosolic cholesterol levels in NPC1 patient fibroblasts [76]. Additionally, the lysosomal Ca^2+^ channel two-pore channel 2 (TPC2) regulates cholesterol accumulation [77]. An agonist of TPC2 (TPC2-A1-P) decreased cholesterol levels with the exocytosis of lysosomes in the human fibroblasts of NPDC patients [77]. The treatment of TPC2-A1-P attenuated the intensity of filipin, the cholesterol-detecting dye, and the fusion of LAMP1 with the plasma membrane [77]. Thus, regulation of the lysosomal proteins has been suggested as a therapeutic strategy for NPDC. However, the activation of lysosomes against NPDC does not always reflect satisfied results. For example, treatment with Torin1, which recovers lysosomal membranes as described above, failed to decrease cholesterol accumulation in lysosomes [75]. Thus, new strategies are needed, and this theme will be addressed in Section 4. Table 1 represents the summary of lysosomal proteins inducing NPDC.

## 3. NPDC in Other Intracellular Organelles

The Golgi is involved in cholesterol trafficking by interacting with other intracellular compartments, including endosomes, lysosomes, and the ER [14,86,87]. The overexpression of endosomal proteins rab7 and rab9 mediated the recovery of BODIPY-LacCer fluorescence, which is a Golgi marker, is reduced in NPDC cells [78]. These results indicate that NPDC affects Golgi-mediated cholesterol trafficking. The caveolin-1 traffics from the plasma membrane to Golgi, while mutations of *NPC1* inhibit the localization of caveolin-1 on Golgi [62]. Treatment with the cholesterol transport inhibitor U18666a revealed the mis-localization of the trans-Golgi network (TGN) marker syntaxin 6 [63], with a decrease in the Golgi cholesterol in NPDC cells. PtdIns4P, a component of Golgi vesicular trafficking, accumulated in U18666a-treated Golgi in human embryonal kidney tsA201 cells [64]. NPC1 proteins mediate the ER-to-Golgi membrane interaction [64]. Treatment with U18666a inhibited the low-density lipoprotein (LDL)-mediated secretion of oxysterol-binding protein (OSBP) from Golgi [65]. Thus, OSBP is a potential cholesterol regulator or sensor in Golgi [65]. Cholesterol-bound OSBP is translocated to Golgi in response to cholesterol depletion [65]. In addition, the Golgi complex regulates NPDC-related proteins. The Golgi membrane protein Golgi-associated retrograde protein (GARP) controls cholesterol trafficking through NPC2 proteins [79]. A previous study showed that GARP recruited NPC2 to lysosomes, and the depletion of VPS53, a subunit of GARP, inhibited NPC2 recruitment to lysosomes and subsequently induced lysosomal cholesterol accumulation [79].

Mitochondria interact with lysosomes to transfer cholesterol, and the activation of mitochondria induces lysosomal activation [80,88]. Stimulation of the mitochondrial chaperone tumor necrosis factor receptor-associated protein1 (TRAP1) increased intracellular ATP levels and activated monophosphate-activated protein kinase (AMPK) [80]. The activation of AMPK inhibited mTORC1, which induced lysosomes to transfer cholesterol from lysosomes to the ER [80]. Knock-down of *NPC1* induced filipin intensity in mitochondria, which triggered oxidative stress in CHO cells [66]. NPC protein deficiency impairs mitochondrial activity. For example, the *NPC^D1005G^* mutation induced decreased mitochondrial volume in Purkinje cells [67], and mitochondrial respiration was reduced in *NPC2*-deficient 3T3-L1 adipocytes [69], *NPC2*-deficient hepatic stellate cells [70], and NPDC patient fibroblasts [68]. Treatment with U18666a and the knock-out of *NPC1* reduced mitochondria-generated ATP and subsequently mediated apoptosis in mice brains [45,71].

NPC1 regulates ER–endolysosome tethering by interacting with the ER sterol transport protein Gramd1b [89]. The deletion of *NPC1* and knock-down of *Gramd1b* decrease the ER–endolysosome contact and increase filipin intensity in HeLa cells [89]. ER-expressed oxysterol-binding protein-related 5 (ORP5) binds with lysosomal NPC1 to transfer cholesterol from the ER to lysosomes [90]. The ER and NPDC complexes are involved in ER functions and cholesterol trafficking. Encarnacao et al. demonstrated that the RNA sequencing of NPDC patient genes revealed the up-regulation of ER stress-related genes, and the *NPC1^R518W^* mutation delayed cholesterol trafficking from the ER to lysosomes [72]. In addition, NPDC cellular models generated by the knock-down of *NPC1* or treatment with U18666a showed the inhibition of the ER–lysosome interaction in CHO cells [72]. Disrupted Ca^2+^ signaling from the inositol 1,4,5-trisphosphate receptor (IP_3_R), which is expressed in the ER membrane, was measured in *NPC1*^I1061T^-mutated human fibroblasts, with reductions in resting ER Ca^2+^ concentrations [73]. Table 1 represents the NPDC in other intracellular organelles.

## 4. Current Therapeutic Strategies for NPDC

**Figure 3:**
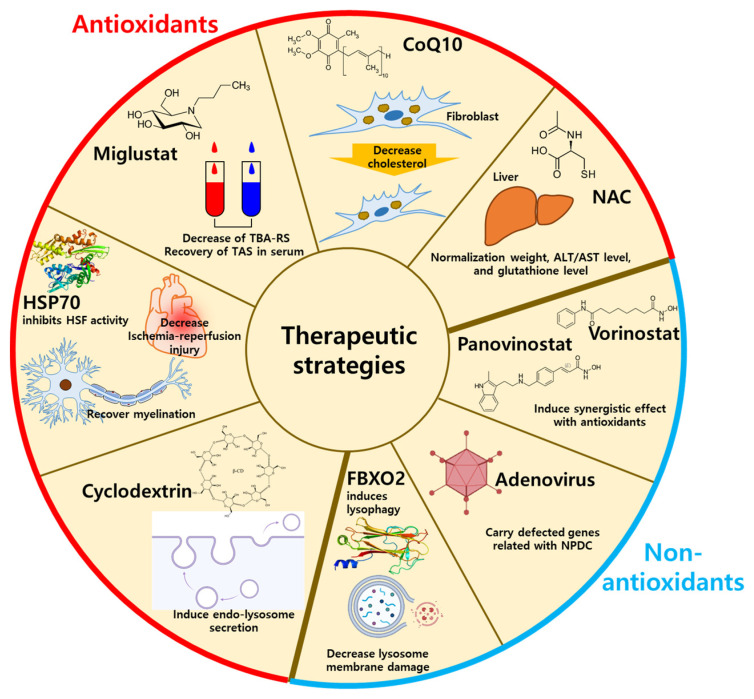
The therapeutic strategies using antioxidants or non-antioxidants. The antioxidants-related NPDC treatment includes HSP70, miglustat, CoQ10, cyclodextrin, and NAC by decreasing oxidative stresses. The non-antioxidant-related NPDC treatment includes lysophagy, panovinostat, vorinostat, and adenovirus by supplementation of antioxidants. HSP70: heat shock protein 70; CoQ10: coenzyme Q10, NAC: *N*-acetylcysteine; FBXO2: F-Box protein 2.

Various approaches to treating NPDC have been studied, such as simply lowering cholesterol, alternatively using other LSD drugs, and manipulating NPDC-related genes [29]. Additionally, adeno-associated viral vectors, heat shock protein (HSP), synthetic high-density lipoprotein (sHDL), histone deacetylase inhibitors, and F-Bod protein2 (Fbxo2)-mediated lysophagy have been suggested as NPDC therapies [91,92,93,94,95]. Especially, sHDL showed high efficiency in reducing cholesterol with cellular safety in *NPC1^I1061T^*-mutated human fibroblasts [92]. Mechanistically, sHDL modulates cholesterol regulatory genes, including *3-hydroxy-3-methylglutaryl-CoA reductase*, *hydroxymethylglutary-CoA synthase*, *ATP binding cassette subfamily A1*, and *NPC1* [92]. Although numerous drugs have been introduced, antioxidant-related drugs and strategies have also been suggested for NPDC treatment. Thus, we summarized the antioxidant-related drugs and non-antioxidant methods for NPDC treatment (Figure 3).

### 4.1. Antioxidant-Related Drugs for NPDC Treatment

#### 4.1.1. *N*-Butyl-Deoxynojirimycin (Miglustat)

As mentioned in the Introduction, oxidative stress is an outcome of NPDC. For example, thiobarbituric acid-reactive species (TBARS), the product of lipid peroxidation, was found to be increased, and the total antioxidant status (TAS) was decreased in the plasma of NPC patients [96]. Although the damaging effects of miglustat, the inhibitor of glycosphingolipid synthesis, on the neuronal system in NPDC patients have not been demonstrated, treatment with miglustat attenuated TBARS levels and recovered TAS levels in the serum of patients with NPDC [96].

#### 4.1.2. *N*-Acetylcysteine and Coenzyme Q10

Antioxidants and antioxidation-related compounds, including *N*-acetylcysteine (NAC) and coenzyme Q10 (CoQ10), have therapeutic effects on NPDC [97]. The ROS scavenger NAC has been suggested as a clinical application to manage oxidative stress [98]. Treatment with NAC recovered liver pathology in NPDC mice [99]. NAC normalized liver weight, plasma levels of alanine aminotransferase (ALT)/aspartate aminotransferase (AST), and liver glutathione levels in NPDC mice [99]. CoQ10 is a component of the electron transport chain and acts as a lipophilic antioxidant [100]. Treatment with CoQ10 decreased cholesterol levels in NPDC patient dermal fibroblasts and cytokine levels in NPDC patient fibroblast-culture media [101].

#### 4.1.3. Heat Shock Factor

Heat shock factors (HSFs) are metabolic proteins that induce protein folding, inhibit protein misfolding and aggregation, and prevent apoptosis [102,103] and oxidative stress [104]. For instance, heat shock protein 70 (HSP70) inhibited the activity of HSF, which induced the antioxidant reaction and reduced oxidative stress and ischemia/reperfusion injury [104,105]. HSP70 also recovered myelination in *NPC* knock-out mice [91,106]. In NPDC, myelin expression is defective because cholesterol is a component of myelination [107]. Thus, myelin defects are considered a marker of NPDC, and HSP70 is suggested for NPDC therapy.

#### 4.1.4. Cyclodextrin

The β-cyclodextrin (CD) is the drug most frequently used for NPDC to decrease lysosomal cholesterol levels [108,109]. β-CD has been suggested as a secondary antioxidant, which enhances traditional antioxidants, such as resveratrol, pterostilbene, pinosylvin, oxyresveratrol, and astaxanthin [110], and β-CD shows antioxidant activity, although its efficacy is lower than that of traditional antioxidants [111]. Although the specific mechanisms of β-CD on lysosomes have not been demonstrated, derivatives of β-CD, such as 2-hydroxypropyl-β-CD (HP-β-CD) and 6-O-α-maltosyl-β-CD (Mal-β-CD), modulate lysosomes. HP-β-CD decreased lysosomal accumulation with decreases in lysosomal sphingolipid in *NPC1*-deleted CHO cells [112]. In HeLa cells, HP-β-CD induced endolysosomal secretion by activating transient receptor potential mucoplin-1 Ca^2+^ channels to reduce accumulated cholesterol [113]. Treatment with HP-β-CD induces lysosome-ER connections to trigger cholesterol trafficking [114]. In the same way, Mal-β-CD decreased the accumulation of lysosomes by reducing lysosomal expansion in *NPC1*-deficient CHO cells [115,116].

### 4.2. Non-Antioxidant Methods for NPDC Treatment

#### 4.2.1. Lysophagy

Lysosomal membrane impairment induces oxidative stress [117,118]. Deletion of the *Fbxo2* gene decreased lysophagy in mouse cortical neurons [94]. Fbxo2 treatment for NPDC decreased lipid-mediated lysosomal membrane damage [94]. These results suggest that oxidative stress is associated with cholesterol trafficking, and thus, lysophagy might help reduce cholesterol accumulation. Although non-antioxidant mechanisms are not the same as those of antioxidants, non-antioxidants effectively assist antioxidant-related drugs through synergistic effects with the development of endosomal and lysosomal trafficking. Thus, we summarized the basic mechanisms of non-antioxidant methods for NPDC and suggested potential applications with antioxidant-related drugs.

#### 4.2.2. Histone Deacetylase Inhibitors

The histone deacetylase inhibitors vorinostat and panobinostat recovered mutated *NPC1^I1061T^* localization [119]. *NPC1^I1061T^* mutation separated NPC1 from lysosomes, and treatment with vorinostat and panobinostat induced the movement of the *NPC1 ^I1061T^* mutant type to lysosomes [119]. Although vorinostat and panobinostat are not antioxidants, the combination of these and antioxidants, such as vitamin C and naringenin, showed synergistic effects with vorinostat and panobinostat [120,121]. Antioxidant-related molecules, including β-CD, vorinostat, and panobinostat, have therapeutic potential for NPDC, but the relationship between these molecules and oxidation has not been fully demonstrated. The Food and Drug Administration (FDA) approved another histone deacetylase, valproic acid (VPA), to reduce cholesterol accumulation in NPDC patient-derived fibroblasts [93]. The combined administration of VPA with another CD-type of M-β-CD induced NPC1 trafficking from endosomes to the ER or Golgi in *NPC1*^I1061T^-mutated human fibroblasts [93], providing evidence of the synergistic effect with antioxidants of lysosomal trafficking.

#### 4.2.3. Adenovirus

Adenovirus has been used as a gene delivery vector to easily penetrate cellular membranes and transfer to the nucleus [122,123]. The transduction of adenovirus cloning with *NPC1* genes induced the stable expression of NPC1 proteins in *NPC1* knock-out mice [124]. Injection with *NPC1*-inserted adenovirus increased the survival rate of Purkinje cells in *NPC1* knock-out mice [125]. Recovery of Purkinje cells by adenovirus injection ameliorated impaired behavior and the survival rates of *NPC1* knock-out mice [125]. Adenovirus early region 3 (RIDα) mimics the role of rab7 protein in the translocation of endosomes and lysosomes [126,127]. Rab7 interacts with lysosomal motor dynein and OSBP family ORP1L, which senses cholesterol levels in lysosomes for the translocation of lysosomes [128,129]. RIDα is substituted for rab7 in rab7-depleted cells to translocate cholesterol [126,127]. RIDα also induced the repositioning of lysosomal and autophagic vesicles to juxtanuclear regions to trigger autophagic flux and cholesterol trafficking in NPDC fibroblasts [130].

## 5. Conclusions and Perspectives

In this review, we demonstrate the relationship between NPDC and intracellular organelles to treat NPDC through the interpretation of antioxidants. NPDC is a lysosomal storage disease with deficient intracellular organelles, including lysosomes, Golgi, ER, and mitochondria. NPDC and each intracellular organelle mutually influence each other. The deficiency of NPC modulates the protein stability and functions of intracellular organelles such as the lysosome, Golgi, ER, and mitochondria. In NPDC patients, cholesterol accumulates in lysosomes and is not transferred to other organelles. Although the direct relationship between NPDC and the antioxidant-related pathway needs to be studied more, intracellular organelles are associated with oxidative stress, and antioxidant reagents have the potential to treat NPDC. Various therapeutic strategies are proposed. For instance, Mglustat, CoQ10, HSP70, and cyclodextrin showed therapeutic effects on NPDC. In addition, sulforaphane, which is enriched in broccoli, induced antioxidative responses in NPDC fibroblasts [131]. The ROS level of NPDC fibroblasts is higher than that of wild-type fibroblasts, and sulforaphane attenuates increases in ROS levels in NPDC [131]. The effect of sulforaphane on NPDC treatment or cholesterol trafficking has not been demonstrated. However, this study showed direct evidence that oxidative stress is related to NPDC. As well as NPDC, other lysosomal storage disorders, including cystinosis and Fabry disease, are related to oxidative stress [132,133], and antioxidants including resveratrol and catechin are suggested for the treatment of mucopolysaccharidosis VII and Gaucher disease [134,135]. In addition, antioxidant Ambroxol enhances β-glucosidase enzyme activity in Gaucher disease patient fibroblasts [136]. Thus, further investigations into the relationship between cholesterol trafficking in NPDC and antioxidants should be conducted and are challengeable issues for the development of therapeutic agents.

## Figures and Tables

**Table 1:** Relationship between NPDC and intracellular organelles.

Induction of NPDC		Organelle Appearance	Strain and Cell Types	Ref.
Lysosome	*NPC1* knock-out	Destruction of lysosomal morphology	NPDC mice platelets	[52]
		Increase in LAMP-1	NPDC patients	[53,54]
		Hyperglycosylation of LAMP-1	*NPC1* knock-out mice	[55]
	U18666a	Decrease in lysosomal Ca^2+^ release	MEG-01	[52]
			RAW 264.7	[58]
		Accumulation of Ptdlns4P and PI4K	NPDC patient fibroblasts	[61]
Golgi	Mutation of *NPC1*	Mis-localization of caveolin-1	Mouse embryonic fibroblasts	[62]
	U18666a	Mis-localization of TGN marker syntaxin 6	CHO cells	[63]
		Accumulation of Ptdlns4P	Human embryonal kidney tsA201 cells	[64]
		Inhibition of OSBP secretion	NPDC fibroblasts	[65]
Mitochondria	Knock-down of *NPC1*	Cholesterol accumulation in mitochondria with oxidative stress	CHO cells	[66]
	Mutation of *NPC1*	Decrease in mitochondrial volume	Purkinje cells	[67]
	*NPC2* deficiency	Decrease in mitochondrial respiration	3T3-L1 adipocytes, hepatic stellate cells, and NPDC patient fibroblasts	[68,69,70]
	U18666a	Decrease in ATP generation with apoptosis	Mice brains	[45,71]
ER	*NPC1* mutation	Up-regulation of ER stress-related genes	CHO cells	[72]
		Disruption of IP_3_R	Human fibroblasts	[73]
	Knock-down of *NPC1*	Inhibition of ER-lysosome fusion	CHO cells	[72]
	U18666a			
Lysosome	Knock-out of *BORC* and *ARL8*	Accumulation of cholesterol in lysosomes	Hela cells	[74]
	Inhibition of mTORC1 by Torin1	Recovery of mitochondrial damage and lysosomal membrane damage	*NPC1* knock-out in mouse embryonic fibroblasts	[75]
	Activation of TFEB by genistein	Increase in LC3 II expression	NPDC patient fibroblasts	[76]
	Activation of TPC2	Decrease in lysosomal cholesterol level by exocytosis	*NPC1*-mutated human fibroblasts	[77]
Golgi	Overexpression of rab7 and rab9	Recovery of Golgi marker	Human skin fibroblasts	[78]
	Depletion of VPS53	Inhibition of NPC2 recruitment	CHO cells	[79]
Mitochondria	Stimulation of TRAP1	Activation of AMPK and inhibition of cholesterol trafficking from lysosomes to ER	Mouse NPDC cells	[80]

Abbreviation: NPDC; Niemann-Pick disease type C, NPC1; Niemann-Pick type C 1, LAMP-1; lysosomal-associated membrane protein-1, PtdIns4P; phosphatidylinositol 4-phosphate, PI4K; phosphatidylinositol 4 kinases, BORC; biogenesis of lysosome-related organelles one-related complex, mTORC1; mammalian target of rapamycin complex 1, TFEB; nuclear translocation of transcription factor EB, TPC2; two-pore channel 2, TGN; trans Golgi network, CHO; Chinese hamster ovary, OSBP; oxysterol-binding protein, IP_3_R; inositol 1,4,5-trisphosphate receptor.
